# Bond Behavior of Steel Bars in Concrete Confined with Stirrups under Freeze–Thaw Cycles

**DOI:** 10.3390/ma15207152

**Published:** 2022-10-14

**Authors:** Guirong Liu, Xiaoxue Dou, Fulai Qu, Pengran Shang, Shunbo Zhao

**Affiliations:** 1 School of Civil Engineering and Communication, North China University of Water Resources and Electric Power, Zhengzhou 450046, China; 2 International Joint Research Lab for Eco-Building Materials and Engineering of Henan, Zhengzhou 450045, China; 3 Collaborative Innovation Center for Efficient Utilization of Water Resources, Zhengzhou 450046, China

**Keywords:** freeze–thaw cycles, stirrup, bond strength, concrete, damage coefficient

## Abstract

In order to evaluate the influence of freeze–thaw action on the durability of concrete structures, this paper presented an experimental study to investigate the effects of freezing–thawing cycles and concrete strength on the bond behavior between steel bars and concrete confined with stirrups. Through freeze–thaw cycles and center pullout tests, the failure mode of pullout specimen, concrete strength, mass loss, dynamic elastic modulus, and bond–slip curves were analyzed. At last, the bond–slip constitutive model was proposed for specimens with stirrup confinement under freeze–thaw action. Main test results indicate that the failure mode and shape of bond–slip curves are affected by stirrups. The bond strength hasa certain increase after 100 freeze–thaw cycles owing to the constraining force from stirrups, whereas the splitting tensile strength significantly declines. After 100 freeze–thaw cycles, the splitting tensile strength of C20 and C40 decreased by 40.8% and 46.5%, respectively. The formula was provided to calculate the bond strength of constrained concrete after freeze–thaw cycles, and the damage coefficient and other related parameters in the formula were suggested. The predicted bond–slip curves are close to the experimental results, which could provide reference for the related research of bond performance after freeze–thaw action.

## 1. Introduction

Concrete has the advantages of high compressive strength, convenience in construction, relatively low cost, and so on, which has been used as the main building material worldwide. However, there exista large amount of tiny pores in concrete, and it is easy to crack. Therefore, water and aggressive ions would penetrate into concrete, decreasing the durability of concrete structures [1,2]. Freeze–thaw action is a major factor in the damage of reinforced concrete structures (especially for dams, aqueducts, inverted siphons, tunnels, and sluices) in cold regions [3,4,5]. Therefore, research on the frost resistance of concrete is of great significance to the durability evaluation and life prediction of reinforced concrete structures [6,7,8,9]. The hydrostatic pressure theory proposed by T.C. Powers believed that the volume expansion of water in concrete pores after freezing at low temperature would cause tensile stress around the concrete pores, resulting in cracks and changes in the internal structure of concrete [10,11]. The existing research results have shown that the influence of freeze–thaw cycles on the strength characteristics of concrete is obvious [12,13]. With the increase in freeze–thaw cycles, the strength of concrete commonly decreased with varying degrees. In addition, the corresponding damage degradation model was put forward according to the experimental results [14,15,16,17].

Bond performance is a key factor for steel bars and concrete working together, which allows longitudinal forces to transfer from steel bars to the surrounding concrete. Bond failure should be avoided in the design or repair of reinforced concrete structures [18,19,20]. It is generally believed that the bond resistance between deformed reinforcement and concrete consists of three parts: the mechanical interaction, friction force, and chemical adhesive [21,22]. Among these three parts, the first two parts account for a large proportion, and the chemical adhesive is very small. Due to the fact that the mechanical interaction and friction force are related to the strength of concrete, concrete strength has a great influence on bond strength. Previous experimental research has studied the effect of concrete strength on bond strength and concluded that the bond strength significantly increased with the increase in compressive strength or splitting tensile strength [23,24,25].

At present, some studies [26,27,28,29] have been carried out on the bond between reinforcement and concrete under the action of freeze and thaw. Experimental results demonstrated that the bond behavior was severely deteriorated for the reason of concrete damage caused by freeze–thaw action. The bond strength obviously decreased, and the corresponding slip increased under the condition of freeze–thaw cycles [30]. Tu et al. [31] investigated the effect of aggregate types on bond performance after frost damage and found that the thermal insulation aggregate was beneficial for the bond behavior and mechanical properties compared with the normal concrete. Xu et al. [32] studied the bond properties of deformed steel bars in concrete subjected to frost damage under monotonic and cyclic loading. The initial bond stiffness and ultimate and residual bond strength were analyzed based on the experiment results. To improve the properties of concrete subjected to freeze–thaw damage and rebar corrosion, Fan et al. [33] studied the effects of nanokaolinite clay on the compressive strength, corrosion condition, and bond properties. While the applications of recycled aggregate concrete have been proposed, many scholars [34,35,36,37] investigated the influence of recycled aggregate on bond behavior in freeze–thaw environments. It is widely accepted that the replacement ratios of recycled coarse aggregate have a great effect on the bond behavior. The bond strength decreased and the slippage increased with the replacement rate of recycled coarse aggregate.

In addition, studies of the effects of stirrups on bond behavior between different types of bars and concrete were conducted by researchers [38,39]. The experimental and analytical results indicated that the stirrup could maintain the constraint effect on the reinforcement after the splitting crack occurred, thereby increasing the bond strength [40,41]. Zheng et al. [42] also found that the effect of stirrups on bond strength was more obvious than that of the concrete cover thickness. Under the condition of stirrup confinement, Qian et al. [43] have carried out experiments of bar location on the bond–slip responses. The influence of stirrups was mainly reflected by the cross-sectional area and spacing of stirrups [44,45,46]. According to the test results of Xu [21,47], the bond strength was proportional to the stirrup cross-sectional area and inversely proportional to the stirrup spacing. However, the variation of bond behavior between reinforcement and concrete confined with stirrups after free–thaw damage is not well-understood.

In this paper, the effect of freeze–thaw cycles on the bond behavior between steel bars and concrete confined with stirrups was experimentally investigated. The failure mode of pullout specimens, bond–slip curves, and concrete strengths were analyzed. Furthermore, the bond strength of steel bars in concrete confined with stirrups under freeze–thaw action was predicted based on the damage coefficient of the concrete. Finally, the bond–slip constitutive model was developed. The present research could be beneficial for the residual structural capacity and repair of reinforced concrete structure in severe cold regions.

## 2. Experimental Program

### 2.1. Specimen Design

**Figure 1:**
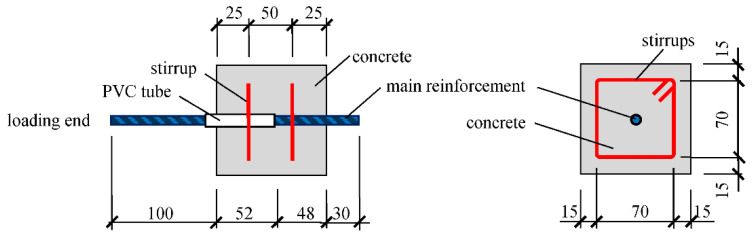
Layout of steel bars and size of bond specimen (size: mm).

In this study, a total of ten group pullout specimens were prepared to investigate the bond properties considering the effects of concrete compressive strengths and freeze–thaw cycles. Every group consists of three specimens; thus, thirty pullout specimens were prepared. The details of the pullout specimens are shown in Figure 1. According to the standard GB/T 50081-2019 [48], the main reinforcement is embedded in the center of the concrete prism. Considering the space of the freeze–thaw testing machine, the pullout specimens were designed as cubes with side lengths of 100 mm.

One HRB500 steel bar with a diameter of 12 mm was used as the main reinforcement in the center, and two HPB300 stirrups with a diameter of 6 mm were arranged to consider the influence of the stirrup constraint. The bonding length was set as four times of the diameter of the main reinforcement, and a PVC tube was embedded in the loading end to reduce the influence of the local stress on the bond. In addition, a total of sixty cubic specimens (100 mm × 100 mm × 100 mm) were cast to measure the compressive strength and splitting tensile strength, and six prism specimens (100 mm × 100 mm × 400 mm) were cast to measure the mass loss and relative dynamic elastic modulus [49].

### 2.2. Materials

Two kinds of concrete with different concrete strength were adopted in this experiment. The mix proportions are shown in Table 1. Ordinary Portland cement P. O 42.5 was used as the cementitious material. The fine aggregate was river sand with a fineness modulus of 2.6, and the natural coarse aggregate was crushed stone with a particle size of 5–20 mm. A naphthalene superplasticizer was added in C40 series of specimens. The water–binder ratios for two series of specimens were 0.60 and 0.44.The measured 28 d compressive strength of concrete is 20.20 MPa and 46.44 MPa. The air content measured in concrete is 4.5%.

The mechanical properties of the two steel bars used in the specimen are shown in Table 2.

### 2.3. Freeze–Thaw Test

**Figure 2:**
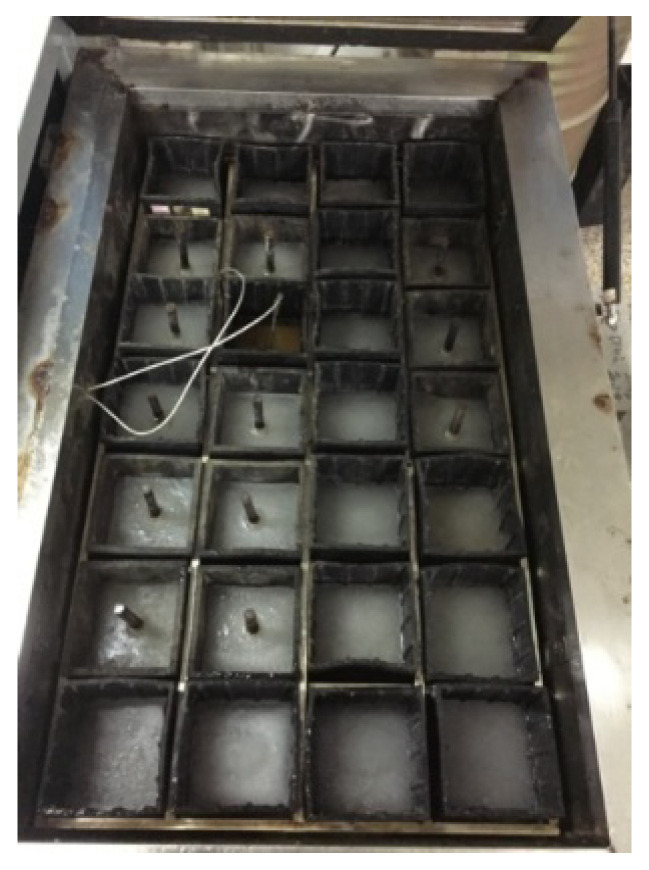
Freeze–thaw test for specimens.

The specimens of the freeze–thaw test were cured at a temperature of 20 °C and 95% relative humidity. Then these specimens were immersed in water for 4 days before the freeze–thaw tests. A quick-freeze method was used according to the standard for test methods of long-term performance and durability of ordinary concrete (GB/T 50082-2009 [49]), as shown in Figure 2.

The temperatures measured in the center of the concrete prism specimen (100 mm × 100 mm × 400 mm) ranged from −20 °C to 7 °C, and each freeze–thaw cycle took 3 h.

### 2.4. Pullout Tests

**Figure 3:**
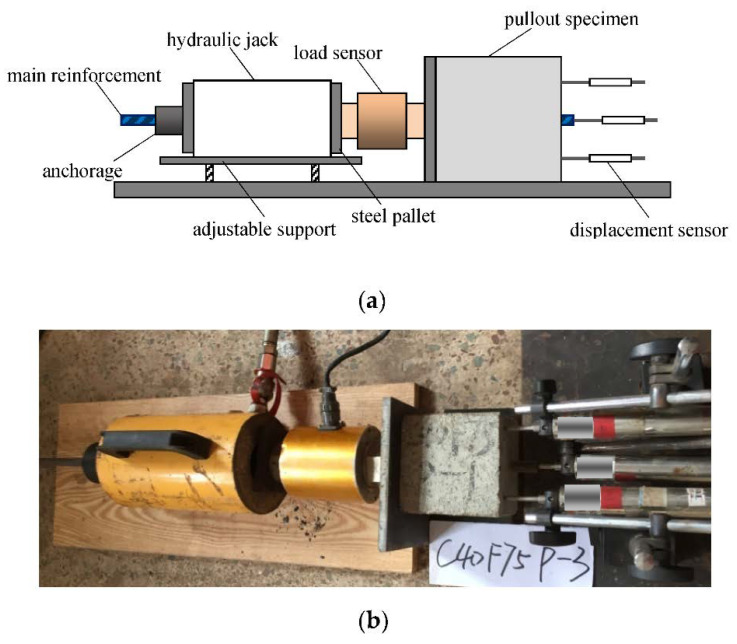
Test setup and instruments for central pullout tests. (**a**) Schematic diagram; (**b**) Reality device.

Central pullout tests were carried out on bond specimens after the freeze–thaw test. Three displacement sensors were arranged at the free end to measure the displacement of concrete and main reinforcement, and a load sensor was arranged at the loading end to measure the bond force, as shown in Figure 3.

The pullout tests were conducted by loading and displacement control. Before reaching the maximum bond force, the loading was controlled at a rate of 7.5 kN/min. When reaching the maximum bond force, the loading rate was controlled at a rate of 0.2 mm/min until the slip reached 15 mm. During the loading process, the load and displacement data were automatically collected and stored by a data acquisition system. The bond stress can be calculated as follows:

(1) $$\tau =\frac{F}{\pi dl_{a}}$$

where *F* is the pullout force, *d* represents the diameter of the main reinforcement, and *l*_a_ is the anchorage length or the bonding length of the main reinforcement.

## 3. Results

### 3.1. Failure Modes

**Figure 4:**
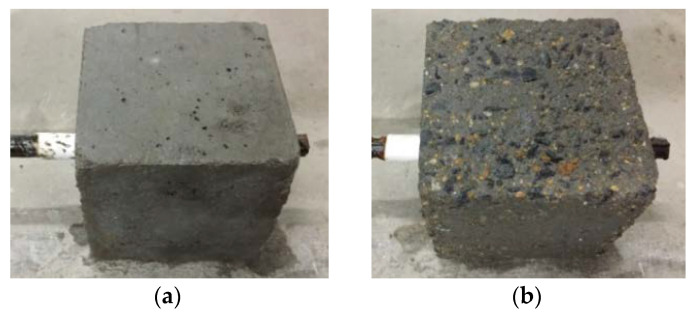
Appearances of concrete before and after freeze–thaw. (**a**) 0 cycleof salt frost; (**b**) 100 cycles of salt frost.

The surface morphology of the specimen significantly changed after the freeze–thaw test, as shown in Figure 4. For the unfreeze–thaw specimen, the surface was smoother and had fewer pores. However, with the increase in freeze–thaw cycles, the pores on the surface of the concrete increased, and the surface concrete spalled. When the freeze–thaw cycles reached 100 times, the concrete surface became rougher and the stones and sands were observed.

**Figure 5:**
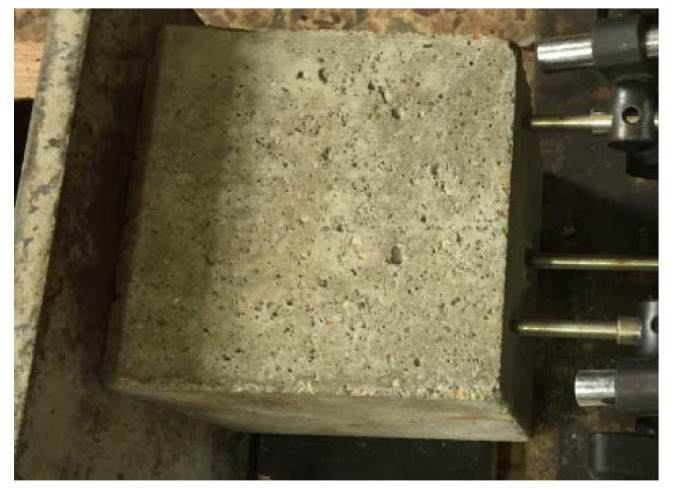
Failure mode of pullout specimen.

Pullout tests were conducted on bond specimens. It was found that the main reinforcements were pulled out for all specimens due to the confinement of stirrups located in concrete, and cracks were not found on the surface of concrete. The pullout failure mode is shown in Figure 5. The failure modes of the bond specimen mainly include splitting failure, pullout failure, and splitting–pullout failure according to the previous studies [39,50,51]. Moreover, the failure mode is affected by the confinement condition, such as the concrete cover and transverse reinforcement. When the specimen was well-confined, the pullout failure mode usually occurred. When the specimen was in unconfined condition, the failure mode was splitting failure instead of pullout failure [52,53].

### 3.2. Concrete Strength

**Figure 6:**
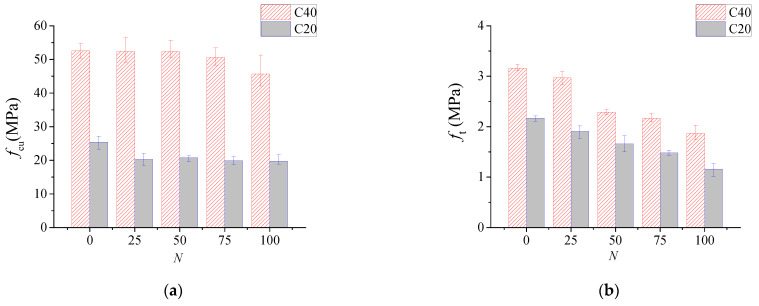
Concrete strengthunder different freeze–thaw cycles *N*. (**a**) Compressive strength; (**b**) splitting tensile strength.

Concrete compressive strength and splitting tensile strength after different freeze–thaw cycles are shown in Figure 6. It can be seen that the concrete compressive strength and splitting strength had a different trend of declining with the increase in freeze–thaw cycles. This conclusion is consistent with the findings of other literature [54,55,56]. After 100 freeze–thaw cycles, the compressive strength of series C40 and C20 decreased to 77.8% and 86.9%, respectively, whereas the splitting tensile strength of series C40 and C20 decreased to 53.5% and 59.2%, respectively. It follows that the loss of splitting tensile strength is higher than that of compressive strength under the same number of freeze–thaw cycles. The reason may be that the micro cracks generated in concrete during the freeze–thaw progress had more unfavorable influence on the splitting tensile strength [57,58].

Based on the linear regression of experimental data, the splitting tensile strength after *N* cycles of freeze–thaw damage could be expressed as follows:

(2) $$f_{t,N}=f_{t,0}\times \left(1-k_{1}\cdot \frac{N}{100}\right)$$

where *f*_t,0_ is the splitting tensile strength before freeze–thaw, MPa; *f*_t,N_ is the splitting tensile strength after *N* cycles of freeze–thaw, MPa; and *k*_1_ represents the coefficient of strength reduction, with *k*_1_ = 0.452 for C20 series and *k*_1_ = 0.426 for C40 series obtained by regression of the test data.

**Figure 7:**
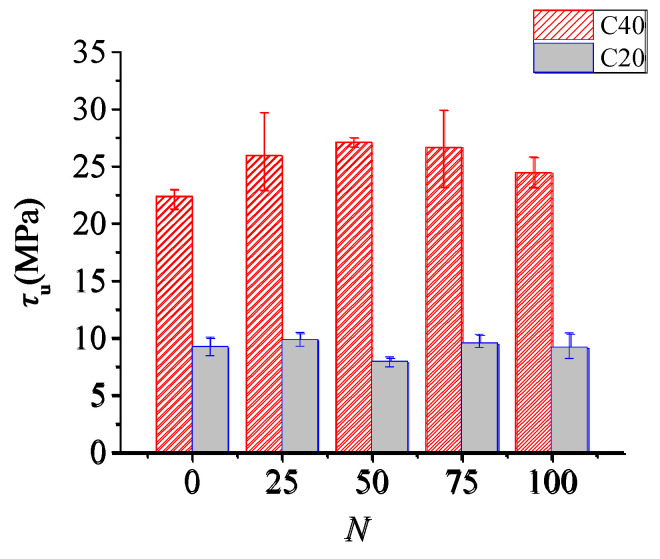
Relationship between ultimate bond strength and *N*.

The variation of bond strength with freeze–thaw cycles is shown in Figure 7. It can be seen that the bond strength of the C20 and C40 series specimens increased at first and then decreased with the increase in freeze–thaw cycles. The bond strength of the C20 specimens after 100 freeze–thaw cycles was reduced by 0.6% compared with that of unfrozen specimens. For the C40 specimens, the bond strength reached the maximum value at 50 freeze–thaw cycles, and the bond strength increased by 9.31% after 100 cycles compared with that of unfreeze–thaw action.

**Figure 8:**
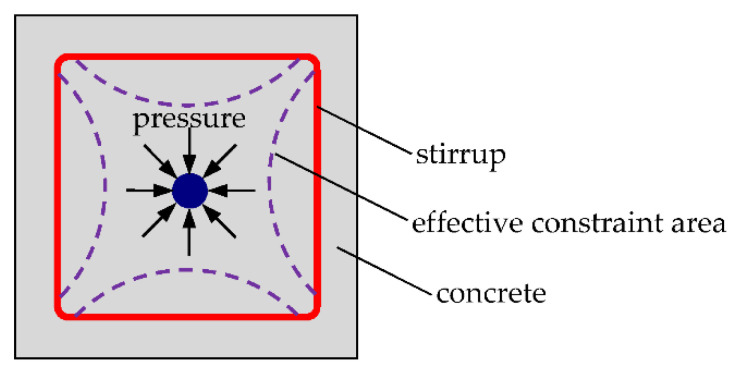
Schematic diagram of stirrup confinement.

Previous studies [54,59] have shown that the bond strength of specimens is proportional to the splitting tensile strength or the square root of the compressive strength of concrete. The splitting tensile strength of concrete decreased with freeze–thaw cycles, resulting in the decrease in the bond strength. However, the bond strength of steel bars in concrete confined with stirrups in this test has an increasing trend. The possible reason for this phenomenon is that freeze–thaw action could cause a certain degree of expansion of concrete [44]. Due to the confinement of stirrups, this expansion trend is constrained, which produces a certain pressure on the steel bars and thus greatly improves the bond strength between steel and concrete, as shown in Figure 8. The confinement effect of stirrups on freeze–thaw concrete improved the bond strength, and its contribution is greater than the “negative effect” of freeze–thaw damage on concrete. As result, the bond strength increases up to 100 freeze–thaw cycles compared with unfrozen specimens. If the number of freeze–thaw cycles continues to increase, the bond strength would decline due to excessive damage of the concrete according to the current trend.

### 3.3. Mass Loss and Dynamic Modulus of Elasticity

Mass loss and dynamic elastic modulus are important parameters to evaluate the damage level of concrete subjected to freeze–thaw cycles. The mass loss of concrete, Δ*W*_N_, is expressed as follows [49]:

(3) $$\Delta W_{N}=\frac{W_{0}-W_{N}}{W_{0}}\times 100$$

where *W*_0_ is the mass of specimens before the freeze–thaw test, *W*_N_ is the mass of specimens after the *N* freeze–thaw test. The mass loss results are summarized in Table 3. It can be found that the maximum mass loss was less than 1.0% for the specimens of C20 and C40 series. This indicates that the air-entraining agent has good effect on the frost resistance of concrete from the perspective of mass loss.

**Figure 9:**
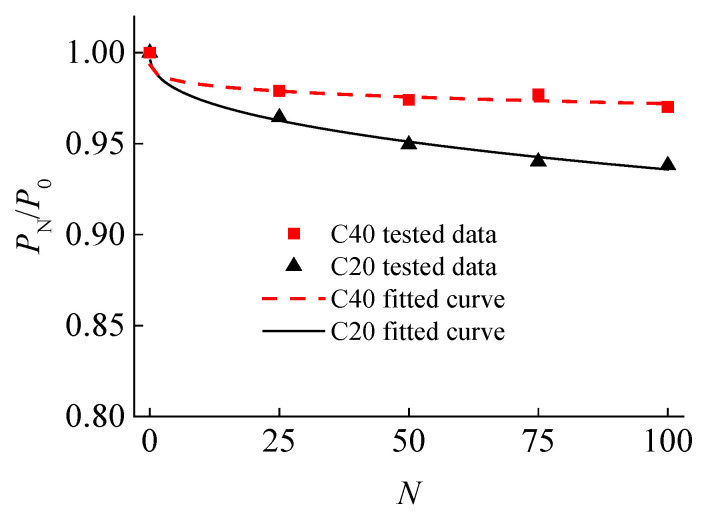
Relationship between normalized relative dynamic elastic modulus and *N*.

There will be a certain degree of damage in concrete under the action of freeze–thaw cycles, thus resulting in the changes of the dynamic elastic modulus [60]. Figure 9 shows the changes of the normalized dynamic elastic modulus with the number of freeze–thaw cycles. It can be found that the relative dynamic elastic modulus decreased with increasing freeze–thaw cycles, which indirectly reflects the damage inside the concrete.

On the basis of concrete damage mechanics, the damage coefficient, *D*_N_, expressed by the relative dynamic elastic modulus [49] was adopted:

(4) $$D_{N}=1-\frac{P_{N}}{P_{0}}$$

where *P*_0_ is the dynamic elastic modulus before freeze–thaw cycles, GPa; and *P*_N_ is the dynamic elastic modulus after *N* freeze–thaw cycles, GPa.

Based on the regression analysis of the experimental data, the relationship between *D*_N_ and *N* was established as follows:

(5) $$D_{N}=1-\alpha N^{\beta }$$

where *N* is the number of freeze–thaw cycles; *α* and *β* are influence parameters, in which the value of *α* is 0.01 for two series of specimens, and the values of *β* for C20 and C40 concrete are 0.39 and 0.20, respectively.

### 3.4. Bond Strength Calculation

For concrete specimens without stirrups, the bond strength decreased with freeze–thaw cycles. The bond strength after freeze–thaw action could be calculated by the splitting tensile strength or compressive strength of concrete [18,19,20,21]. However, for concrete specimens confined with stirrups, the bond strength was generally increased in the range of 100 freeze–thaw cycles studied in this paper. This indicates that, although freeze–thaw causes deterioration in concrete, the compressive stress on the main reinforcement increases due to the expansion of concrete.

Based on the existing bond strength calculation formula of ribbed steel bars in concrete confined with stirrups [47], the following formula was used to calculate the bond strength after freeze–thaw cycles:

(6) $$\tau_{u}=\left(0.82+0.9\frac{d}{l_{a}}\right)\cdot \left(k+0.7\frac{c}{d}+K_{\mathrm{svN}}\frac{A_{\mathrm{sv}}}{c\cdot s_{\mathrm{sv}}}\right)f_{\mathrm{tN}}$$

where *k* is a constant and its value is 0.9; *c* is the concrete cover of the main steel bar; *K*_svN_ is the restraint enhancement factor of stirrups after freeze–thaw cycles; *A*_sv_ is the section area of stirrups; and *s*_sv_ is the spacing of transverse stirrups.

**Figure 10:**
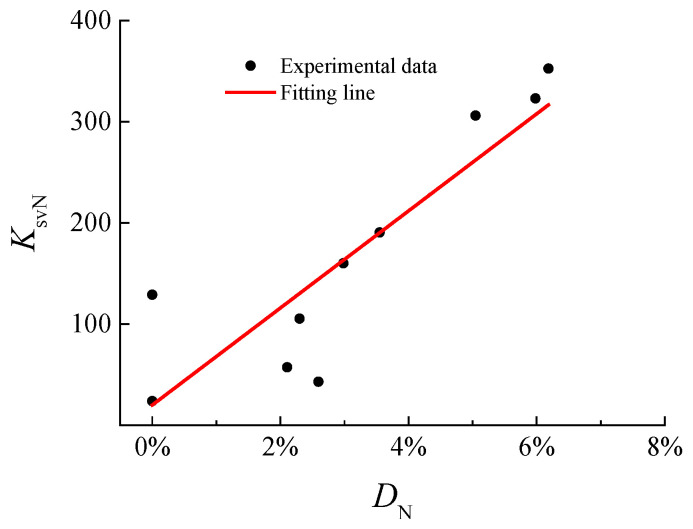
Relationship between *K*_svN_ and *D*_N_.

The restraint enhancement factor of stirrups *K*_svN_ is affected by the number of freeze–thaw cycles, but it is essentially related to the concrete damage induced by freeze–thaw action. The relationship between coefficient *K*_svN_ and *D*_N_ was obtained by the analysis of experimental data, as shown in Figure 10.

The fitting function in this study is shown in Formula (7), with the correlation coefficient of 0.878.

(7) $$K_{\mathrm{svN}}=4854D_{N}+20$$

It can be found that *K*_svN_ is equal to 20 when *D*_N_ = 0, which is the same asthe coefficient value of ordinary concrete without freeze–thaw cycles. According to Formula (7), the bond strength after different freeze–thaw cycles can be calculated. Additionally, the calculated results and experimental bond strengths are listed in Table 4. The average value of the ratio between the calculated values and experimental data of the bond strength is 1.04, and the correlation coefficient is 0.904. The formula given in this paper could be used to predict the bond strength between reinforcement and concrete confined with stirrups subjected to freeze–thaw damage.

### 3.5. Bond–Slip Curves

**Figure 11:**
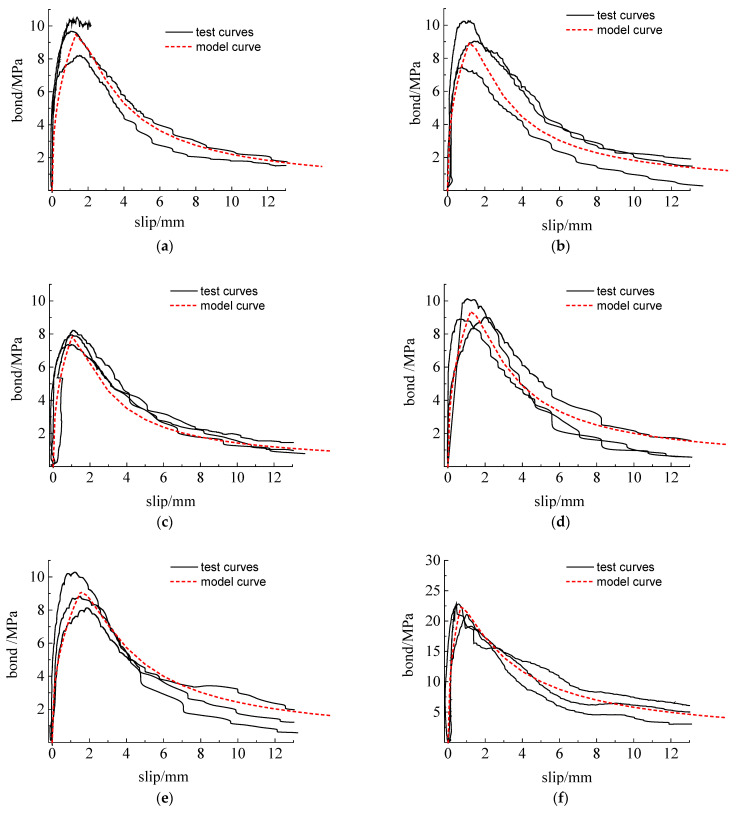

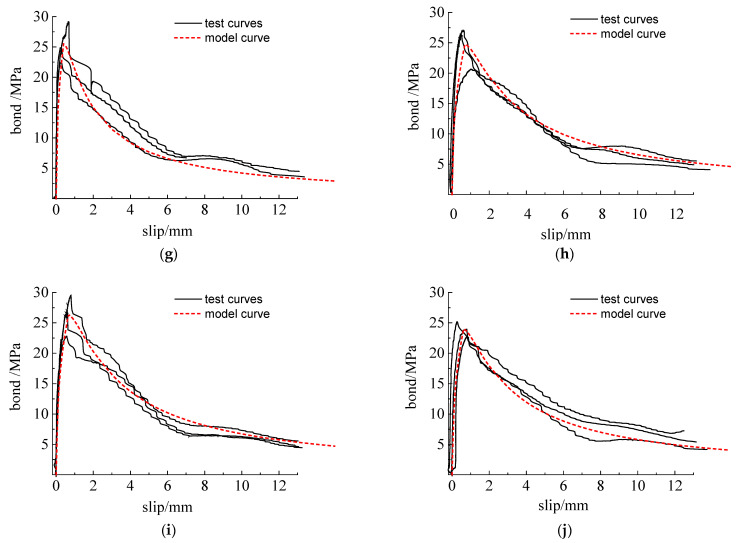
Bond–slip relationship curves ofC20 and C40 specimens with ribbed steel bars. (**a**) C20F0; (**b**) C20F25; (**c**) C20F50; (**d**) C20F75; (**e**) C20F100; (**f**) C40F0; (**g**) C40F25; (**h**) C40F50; (**i**) C40F75; (**j**) C40F100.

Two series of bond–slip curves of specimens with different concrete strength are shown in Figure 11. It can be found that the bond strength and slip were different, but the curve shapes were similar because of the confinement of stirrups. The bond–slip curve between reinforcement and concrete confined with stirrups was composed of the elastic, sliding, descending, and residual stages. For the descending stage, the bond stress slowly dropped due to the confinement of stirrups, which exhibited better ductility. Moreover, the bond strength increased with concrete strength, but the slip at the peak strength correspondingly decreased from about 1.2 mm to 0.6 mm. When the concrete strength is the same, the effect of freeze–thaw cycles on the slip value corresponding to the ultimate bond stress is not obvious.

### 3.6. Bond–Slip Constitutive Model

To analyze the mechanical properties of reinforced concrete structures under a freeze–thaw environment, it is necessary to use the bond–slip constitutive model between reinforcement and concrete. A two-stage model (including ascending stage and descending stage) was adopted, and the bond–slip relationship can be expressed by the following equations [61,62]:

(8) $$y=x^{A},\;\;0\leq s\leq s_{u}$$

(9) $$y=\frac{x}{B{\left(x-1\right)}^{2}+x},\;s>s_{u}$$

where $y=\tau /\tau_{u}$; $x=s/s_{u}$; *s* is the slip between reinforcement and concrete; *s*_u_ is the slip corresponding to the peak bond strength; and A and B are the parameters determined by test results. The value of parameter A could be obtained by nonlinear curve fitting; A = 0.4 was adopted in this study for all groups of specimens considering the suggested value of other literature [63,64].Parameter B was obtained according to the residual bond strength $\tau_{r}$ and the corresponding slip *s*_r_. The expressions of $\tau_{r}$ and $s_{r}$ are given as follows according to GB/T 50010-2010 [65]:

(10) $$\tau_{r}=\tau_{u}/3$$

(11) $$s_{r}=0.55d$$

Based on the regression analysis of the test results, the value of B for C20 and C40 series was obtained as 0.58 and 0.22, respectively. The bond–slip constitutive model was established, and the predicted results are shown in Figure 11. The predicted curves agreed well with the tested curves, which could be used for simulating the bond behavior between reinforcement and concrete confined with stirrups after freeze–thaw action.

## 4. Discussion

Reinforced concrete structures in cold regions are usually subjected to freeze–thaw action, which leads to the deterioration of concrete and the decrease in concrete strength. Since bond performance is the basis for steel bars and concrete working together, the variation of the bond between steel bars and concrete under freeze–thaw action deserves attention. Previous research mainly focused on the bond performance of specimens without stirrups, and the influence of stirrups was not considered. Considering the restraint effect of stirrups on concrete during the process of concrete freeze–thaw damage and its influences on the bond strength and bond–slip curve, this paper has carried out relevant research work.

Through the experimental phenomena of this paper, it was found that the pullout failure occurred for the bond specimens due to the restraint effect of stirrups. This failure mode is different from other studies in which splitting failure or splitting–pullout failure occurs for specimens without stirrups or with small concrete cover [17,63]. In this study, with the increase in freeze–thaw cycles, the bond strength of the C20 group changed little compared with the nonfreeze–thaw specimens. On the other hand, for the C40 group of specimens, the bond strength firstly increased and then decreased, and the bond strength subjected to 100 freeze–thaw cycles did not decrease compared with the control specimens. This may be due to the effect of confinement provided by stirrups on the bond strength during the freeze–thaw progress. In addition, the freeze–thaw damage of concrete, such as the generation and expansion of cracks, may be reduced due to the confinement of the stirrups. However, the current study also found that the bond strength showed a downward trend when freeze–thaw cycles exceeded a certain number of times. This indicates that the negative influence of concrete damage exceeds the positive influence of stirrups.

The relative dynamic elastic modulus could reflect the damage degree of concrete subject to freeze–thaw action; therefore, the damage coefficient *D*_N_, as shown in Equation (4), is used as a variable to determine the restraint enhancement factor of stirrups *K*_svN_ expressed as Equation (7). Then the ultimate bond strength is determined using Formula (6). This formula can be used to evaluate the bond strength between ribbed bars and concrete with or without stirrups subject to freeze–thaw cycles. It is found from the present study that the constraint effect of stirrups mainly affects the descending stage of the bond–slip curve, which is significantly different from the steeper descending stage of the curve of the specimen without stirrups [30]. Based on the existing bond–slip constitutive model, a bond–slip model under stirrup confinement was established. It should be noted that the descending stage of this model is only applicable to specimens with stirrups.

This study has certain reference value for the evaluation of the mechanical performance of reinforced concrete structures under freeze–thaw action and also can help researchers to select the relevant parameters in the bond–slip constitutive model. There are several factors affecting the bond performance between steel bars and concrete, such as the steel bar diameter, bond strength, concrete cover, and stirrup ratio. Due to the limitation of the present study, only part of the research was carried out, and the maximum number of freeze–thaw cycles is set to 100. Therefore, further research should be carried out to investigate the influence of these factors on the bond performance, and the number of freeze–thaw cycles should be increased to obtain more comprehensive rules.

## 5. Conclusions

(1) Due to the confinement effect of transverse stirrups, the generation of cracks was effectively prevented, and steel bar pullout failure was observed for all bond specimens in this study. This is different from the splitting–pullout failure mode of specimens without stirrups. The shape of the bond–slip curve was affected by stirrups in concrete and exhibited better ductility.
(2) The internal pore structure of concrete was destroyed by freeze–thaw damage, which made the compressive strength and splitting tensile strength decrease to some extent. In this experiment, the compressive strength of C20 and C40 concrete after 100 freeze–thaw cycles decreased by 22.2% and 13.1%, respectively. Furthermore, the splitting tensile strength decreased by 46.5% and 40.8%, respectively. Freeze–thaw action had greater influence on the splitting tensile strength than compressive strength of concrete.
(3) Transverse stirrups could effectively improve the bond performance between steel bars and concrete. Under the action of 100 freeze–thaw cycles, the bond strength did not decrease compared with that of nonfreeze–thaw specimens. However, the bond strength had a descending trend based on the experimental data analysis.
(4) On the basis of the existing bond strength model for unfrozen concrete, the formula expressed by the damage coefficient was given for calculating the bond strength after freeze–thaw cycles considering the effects of concrete strength and stirrup confinement. The comparison between calculation and experiment shows that the formula used in this paper can effectively predict the bond strength confined with stirrups subject to freeze–thaw cycles.
(5) The bond–slip constitutive model was developed for deformed steel bars. This model is applicable for specimens with or without stirrups under freeze–thaw action. The relevant parameter values were suggested by fitting the test curves. It was found that the model had good accuracy and could provide references for the calculation of reinforced concrete structures in cold regions.

## Figures and Tables

**Table 1:** Mix proportion of concrete (kg/m^3^).

Specimen Series	Water	Cement	Sand	Stone	Superplasticizer	Air-Entraining Agent
C20	210	350	755	1086	0	0.375
C40	165	375	704	1200	3.375	0.375

**Table 2:** Mechanical properties of steel bars.

Steel Type	Diameter (mm)	Yield Strength (MPa)	Tensile Strength (MPa)	Rate of Elongation (%)
HPB300	6	372.6	566.0	25.3
HRB500	12	619.0	727.5	15.7

**Table 3:** Mass loss of the specimens after freeze–thaw cycles.

Freeze–Thaw Cycles *N*	Weight Loss ΔW_N_/%	
	C20 Group	C40 Group
0	0	0
25	0.13	0.25
50	0.38	0.41
75	0.50	0.36
100	0.11	0.46

**Table 4:** Calculation and experimental values of bond strength.

Group No.	*D*_N_/%	*f*_tN_/MPa	*τ*_u,test_/MPa	*τ*_u,cal_/MPa	*τ*_u,cal_/*τ*_u,test_
C20F0	0	2.17	9.25	9.03	0.976
C20F25	2.11	1.91	9.86	13.18	1.337
C20F50	2.59	1.66	7.93	12.52	1.577
C20F75	2.30	1.48	9.55	10.59	1.110
C20F100	2.98	1.16	9.19	9.34	1.016
C40F0	0	3.16	22.39	13.14	0.587
C40F25	3.55	2.97	25.96	26.09	1.005
C40F50	5.05	2.29	27.12	24.58	0.907
C40F75	5.98	2.17	26.69	25.94	0.972
C40F100	6.19	1.87	24.48	22.85	0.934

## Data Availability

The data of this study are available from the corresponding author upon reasonable request.
